# Development and validation of a novel prediction model to identify patients in need of specialized trauma care during field triage: design and rationale of the GOAT study

**DOI:** 10.1186/s41512-019-0058-5

**Published:** 2019-06-20

**Authors:** Rogier van der Sluijs, Thomas P. A. Debray, Martijn Poeze, Loek P. H. Leenen, Mark van Heijl

**Affiliations:** 1 Department of Traumatology, Maastricht University Medical Center, Maastricht, The Netherlands; 2 Department of Traumatology, University Medical Center Utrecht, Utrecht, The Netherlands; 3 Department of Surgery, Diakonessenhuis Utrecht/Zeist/Doorn, Utrecht, The Netherlands; 4 Julius Center for Health Sciences and Primary Care, University Medical Center Utrecht, Utrecht University, Utrecht, The Netherlands; 5 Cochrane Netherlands, University Medical Center Utrecht, Utrecht University, Utrecht, The Netherlands

**Keywords:** Triage, Trauma Triage App, Prediction model, Emergency Medical Services, Study protocol, Machine learning, Gradient boosting, Meta-analysis, Diagnosis, External validation

## Abstract

**Background:**

Adequate field triage of trauma patients is crucial to transport patients to the right hospital. Mistriage and subsequent interhospital transfers should be minimized to reduce avoidable mortality, life-long disabilities, and costs. Availability of a prehospital triage tool may help to identify patients in need of specialized trauma care and to determine the optimal transportation destination.

**Methods:**

The GOAT (Gradient Boosted Trauma Triage) study is a prospective, multi-site, cross-sectional diagnostic study. Patients transported by at least five ground Emergency Medical Services to any receiving hospital within the Netherlands are eligible for inclusion. The reference standards for the need of specialized trauma care are an Injury Severity Score ≥ 16 and early critical resource use, which will both be assessed by trauma registrars after the final diagnosis is made. Variable selection will be based on ease of use in practice and clinical expertise. A gradient boosting decision tree algorithm will be used to develop the prediction model. Model accuracy will be assessed in terms of discrimination (c-statistic) and calibration (intercept, slope, and plot) on individual participant’s data from each participating cluster (i.e., Emergency Medical Service) through internal-external cross-validation. A reference model will be externally validated on each cluster as well. The resulting model statistics will be investigated, compared, and summarized through an individual participant’s data meta-analysis.

**Discussion:**

The GOAT study protocol describes the development of a new prediction model for identifying patients in need of specialized trauma care. The aim is to attain acceptable undertriage rates and to minimize mortality rates and life-long disabilities.

## Introduction

Prehospital trauma triage is essential to get the right patient to the right hospital [1]. Erroneously transporting a patient requiring specialized trauma care to a lower-level trauma center is associated with higher mortality rates [2, 3]. Conversely, transporting a patient not in need of specialized trauma care to a higher-level trauma center results in extra costs and overutilization of resources. These key metrics for triage quality are termed undertriage and overtriage, respectively. The American College of Surgeons Committee on Trauma guidelines state that trauma systems must aim to attain a maximum of 5% undertriage [1].

One key component in the diagnostic strategy that determines the initial transportation destination is the use of a prehospital triage tool. These tools often involve the use of a prediction model or a flowchart where fulfillment of one of multiple criteria indicates the need for specialized trauma care. Unfortunately, a recent systematic review identified that the discriminative ability of many existing tools is quite poor [4]. One of the reasons is that simplification is key to facilitate their usefulness in clinical practice, thereby degrading predictive accuracy. There is limited time to collect patient data on-scene, and diagnostic modalities are very limited compared to hospitals.

The Trauma Triage App (TTApp) was recently developed to overcome the typical trade-off between simplicity and predictive accuracy. This mobile application implements a logistic regression model to estimate the need of specialized trauma care and provides an easy to use interface. This (reference) model was developed using individual participant’s data (IPD) from a single Emergency Medical Service (EMS) in the Netherlands. When the model was externally validated in a different EMS in the Netherlands, we found an undertriage rate of approximately 11%, at cost of < 50% overtriage [5].

Although the reference model outperformed other tools, its discriminative value and generalizability could potentially be improved using a machine learning algorithm, a greater amount of IPD, participating EMSs and hospitals, and a more robust development strategy [5].

In particular, the relatively small sample size (4950 patients, with 435 patients in need of specialized trauma care) limited the use of interaction terms and non-linear effects for modeling the included predictors and prevented any insight into the model’s generalizability across different EMSs in the Netherlands. Therefore, the aims of the GOAT (Gradient Boosted Trauma Triage) study are (1) to develop a new prediction model on nationwide IPD that accurately identifies patients in need of specialized trauma care in a prehospital setting, (2) to validate this prediction model on IPD from multiple EMSs during development, (3) to investigate sources of heterogeneity in model performance, and (4) to compare it to the reference model used in the initial version of the TTApp.

## Methods/design

### Study design

This is a prospective, multi-site, cross-sectional diagnostic study that is conducted to predict the need of specialized trauma care during field triage. We will adhere to existing recommendations on diagnostic model development, IPD meta-analysis (IPD-MA), and report the resulting model in accordance with the Transparent Reporting of a multivariable model for Individual Prognosis or Diagnosis (TRIPOD) guidelines [6–9]. Data collection started at January 1, 2015, and ended at December 31, 2018.

### Participants

All patients, suspected of injury, transported by a ground EMS from the scene of injury to any emergency department in the Netherlands will be potentially eligible. The Netherlands is divided into 25 different EMS regions and 11 inclusive trauma systems. At least five different EMS regions will be included. These EMS regions have to be representative for urban, suburban, and rural areas. All hospitals, and consequently all trauma systems, with receiving emergency departments in the Netherlands collect the required patient outcomes and participate in this study.

### Data collection

Two distinct data sources will be merged to create a final dataset. These data sources consist of prehospital run reports, collected in a standardized manner by multiple EMSs, and the Dutch National Trauma Registry (in Dutch, *Landelijke Trauma Registratie* [LTR]). Run reports used by included EMSs are based on the template of the Basic Set of Ambulance Care (in Dutch, *Basisset Ambulancezorg* [BSA]) and include demographics, physiological characteristics, mechanism of injury, injuries, patient status, on-scene treatments, initial transportation destination, and more. The LTR is a nationwide registry that collects patient data in accordance with an extended version of the Utstein registry template for uniform reporting of data following major trauma [10]. This registry covers all trauma-related hospital admissions of trauma-receiving emergency departments in the Netherlands since 2015 [11–13]. Relevant patient outcomes included in this registry are, among others, Injury Severity Scores (ISS), early critical resource use, intensive care unit admission, and death. Patient identification numbers used by EMSs are collected when available.

**Fig. 1:**
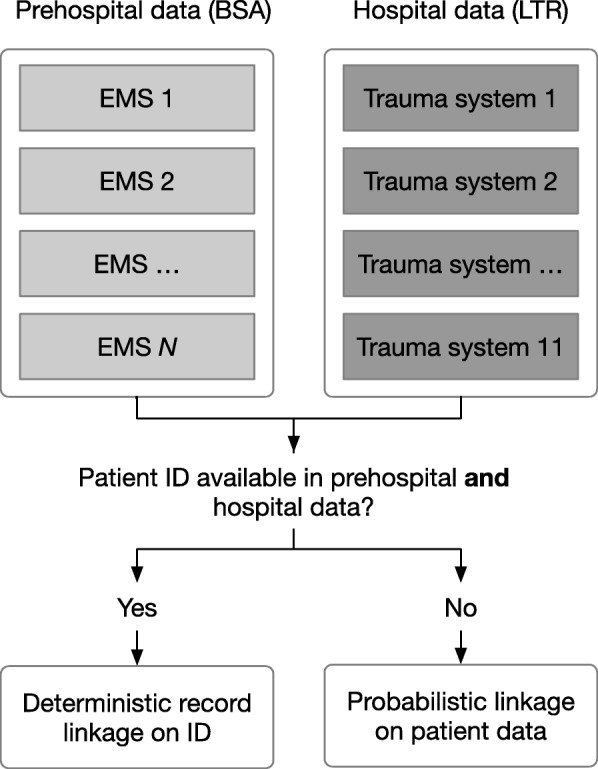
Data collection and record linkage

A combined deterministic and probabilistic linkage scheme will be used to match prehospital run reports and data from the LTR. Records are deterministically linked when prehospital patient identification numbers are available in both datasets. A probabilistic approach will be used to match patient records when unique identifiers are lacking. This approach utilizes machine learning methods and distance functions to identify matching records. Patients discharged directly from the emergency department are presumed not to have any of the investigated patient outcomes. This assumption combined with linking hospital and prehospital records had a sensitivity of 99.7% (95% CI, 99.0–99.9) and specificity of 100.0% (95% CI, 99.7–100.0) in previous studies [14]. The full data collection and record linkage strategy are depicted in Fig. 1.

### Outcome

The primary outcome is an ISS ≥ 16 coded by trained trauma registrars within 30 days after the emergency department admission. This reference standard is based on the Abbreviated Injury Scale version 2005, update 2008, and is recommended by the American College of Surgeons Committee on Trauma to evaluate triage quality [1]. Treating patients with ISS ≥ 16 in higher-level trauma centers is associated with lower mortality rates [2, 15, 16]. The ISS is an anatomical score that is calculated after the final diagnosis is made. Since it is based on anatomic criteria, it is assumed to be identical to the patient status on-scene and is thus used as a diagnostic reference standard.

Because ISS is not perfectly correlated with resource utilization, we included a secondary, resource-based outcome measure to define the need for specialized trauma care [17, 18]. The secondary outcome is early critical resource use, which is a composite endpoint consisting of intubation in the prehospital setting, major surgical intervention, radiological intervention, or death within 24 h, as well as discharge to the intensive care unit from the emergency department. A similar endpoint is used in prior studies on prehospital trauma triage [19].

### Predictor selection

Time is critical during field triage. Therefore, the number and complexity of hand-collected variables must be limited. To prevent the delay of definitive treatment, variables should be easily accessible during routine care, clearly defined, and measured in a standardized and reproducible way to improve transferability and predictive stability [8]. The candidate variables for model development were predefined based on prior evidence and clinical reasoning (Table 1). For instance, many candidate variables are criteria from the Field Triage Decision Scheme, which is the primary triage tool used by EMSs in the US [1]. The final set of variables will be selected prior to model development. The selection of variables is therefore independent of their performance in the training data. Additional predictors (i.e., features), which are not predefined, will be engineered from these variables (e.g., the date of injury might be converted to three predictors indicating the day of the week, the current month, and the current season of the year).

**Table 1:** Candidate variables for predictor engineering

Variable	Reason for inclusion
Demographics	
Age	Included in the FTDS
Gender	Associated with the reference standard in previous research and interacts with other candidate variables
Vital signs	
Glascow Coma Scale, eyes component	Included in the FTDS
Glascow Coma Scale, motor component	Included in the FTDS
Glascow Coma Scale, verbal component	Included in the FTDS
Systolic blood pressure	Included in the FTDS
Diastolic blood pressure	Expected interactions with other candidate variables (e.g., systolic blood pressure)
Heart rate	Expected interactions with other candidate variables (e.g., systolic blood pressure)
Respiratory rate	Included in the FTDS
Intubation	Direct indication of resource use
Oxygen saturation	Associated with the reference standard in previous research and expected interactions with other candidate variables
Mechanism of injury	
MVA (excl. motorcycles, mopeds, scooters)	Included in the FTDS
Motorcycle accident	Included in the FTDS
Moped, scooter accident	
MVA, pedestrian	Included in the FTDS
MVA, different	Included in the FTDS
Gunshot	Expected association with the reference standard and other candidate variables (e.g., penetrating injury)
Stab wound	Expected association with the reference standard and other candidate variables (e.g., penetrating injury).
Struck with blunt object	Expected association with the reference standard
Fall, same level	Included in the FTDS
Fall, higher level	Included in the FTDS
Asphyxia	Associated with the reference standard in previous research
Burns, % of body surface	Associated with the reference standard in previous research
Injury type	
Penetrating injury to head, neck, torso, and extremities proximal to elbow and knee	Included in the FTDS
Flail chest	Included in the FTDS
Paralysis	Included in the FTDS
Open or depressed skull fracture	Included in the FTDS

*Abbreviations: MVA* motor vehicle accident, *FTDS* Field Triage Decision Scheme

The TTApp allows prediction models to use additional variables collected by the device on which the algorithm is embedded. These variables do not delay treatment since collection is computerized. Traveling times, global positioning systems locations, date, and time are variables that might provide extra predictive power to the hand-collected variables. Many predictors can be engineered from these variables, such as the season of the year, day of the week, regions, daytime or night, and more. No constraints are posed on the number and type of predictors that can be derived from these variables during the development phase.

### Missing data

Most prediction modeling methods, such as logistic regression, are not able to deal with missing values and therefore require special care during development, validation, and implementation. For trauma triage, missing values are a particular concern because there may not always be time to measure critical variables. For this reason, we here adopt gradient boosting decision trees for prediction model development, as resulting prediction models can deal with missing values upon implementation. Briefly, decision tree algorithms implement surrogate splits for predictors with missing values and loosely operate under a missing-at-random assumption (as splits are conditional on some of the observed data). This yields an advantage in real-life situations, where prehospital data are often not fully available and surrogate splits can therefore be used to obtain an individual prediction nevertheless.

Multiple imputation will be used to address missing variables in the dataset in order to validate the reference model (which cannot accommodate for missing values). We will adopt multiple imputation methods that account for clustering across sites. Fifty different imputed datasets will be generated using chained equations by the R package MICEMD [20, 21]. Analyses will be applied to each individual dataset. Results will be averaged to provide point estimates. Confidence intervals will be calculated according to Rubin’s rules [22].

### Statistical analysis methods

In this study, we will develop a gradient boosting decision tree with the LightGBM Python library, and we will compare it to the reference model by the means of internal-external cross-validation [23–25].

Boosting is an ensemble technique that involves the estimation of multiple, related, prediction models [26]. The core concept of boosting is to add new models to the ensemble sequentially, in contrast to other ensemble strategies. Each model added to the ensemble is trained with respect to the error of the previously estimated models. Boosting can be applied to various families of prediction models and is often used in conjecture with decision trees [27]. The LightGBM Python library extends the boosting principle with various tunable hyperparameters (e.g., maximum tree depth, number of boosting iterations, custom objective functions) and regularization methods (e.g., subsampling a ratio of columns when constructing a new tree). Furthermore, it deals with missing data by sparsity-aware split finding. The default direction of a node is learned in the tree construction process, so that it minimizes the error in the training data.

A robust model development strategy will be implemented to avoid model optimism. First, internal-external cross-validation (IECV) will be used generate *N* pairs of development and (non-random) validation samples, where *N* is the number of participating clusters (EMSs). This technique iteratively uses IPD from *N–*1 clusters to develop a prediction model and the remaining cluster’s IPD for its external validation. This yields *N* scenarios in which model performance can be investigated in an independent sample and compared to the reference model. A major difference with traditional cross-validation is that hold-out samples in IECV are non-random if the available clusters differ from one another, which allows to assess model generalizability (rather than reproducibility).

In each of the *N* training datasets, we will develop a prediction model using LightGBM. The set of predefined hyperparameters will be optimized for each model using ten iterations of stratified tenfold cross-validation with a shuffle prior to each iteration (see Table 2). Hereto, we will adopt a Tree-structured Parzen Estimator algorithm to minimize the mean squared error within a restricted search space in 500 iterations [28]. We limited the amount of hyperparameters to be optimized to avoid overfitting and to enable more extensive modeling of individual predictors.

**Table 2:** Hyperparameters

Parameter	Explanation
Free	
Learning rate	Shrinkage rate (how much will the weights be adjusted every iteration).
Number of leaves	Maximum number of leaves in one tree.
Lambda L1	L1 regularization.
Lambda L2	L2 regularization.
Feature fraction	Randomly select part of the predictors on each iteration.
Fixed	
Early stopping	The cross-validation score needs to improve at least every n round to continue with the next boosting iteration.
Maximum depth	Maximum tree depth (note that it is less relevant here since the tree grows leaf-wise).
Minimum data	Minimal number of records in one leaf. A higher number prevents overfitting.
Bagging fraction	Randomly select part of the data without resampling.
Bagging frequency	Per how many rounds should bagging be applied.
Unbalanced data	Does data need to be balanced or not.

Second, in each IECV round, we will externally validate the developed model in the test sample and assess its discrimination (c-statistic) and calibration (intercept, slope, and plot) performance. Two scenarios will be explored, one including class weights that are inversely proportional to the outcome occurrence in the development data and one without class rebalancing. We will also assess its comparative performance with the reference model, by quantifying the difference in c-statistic and performing decision curve analysis [29].

**Fig. 2:**
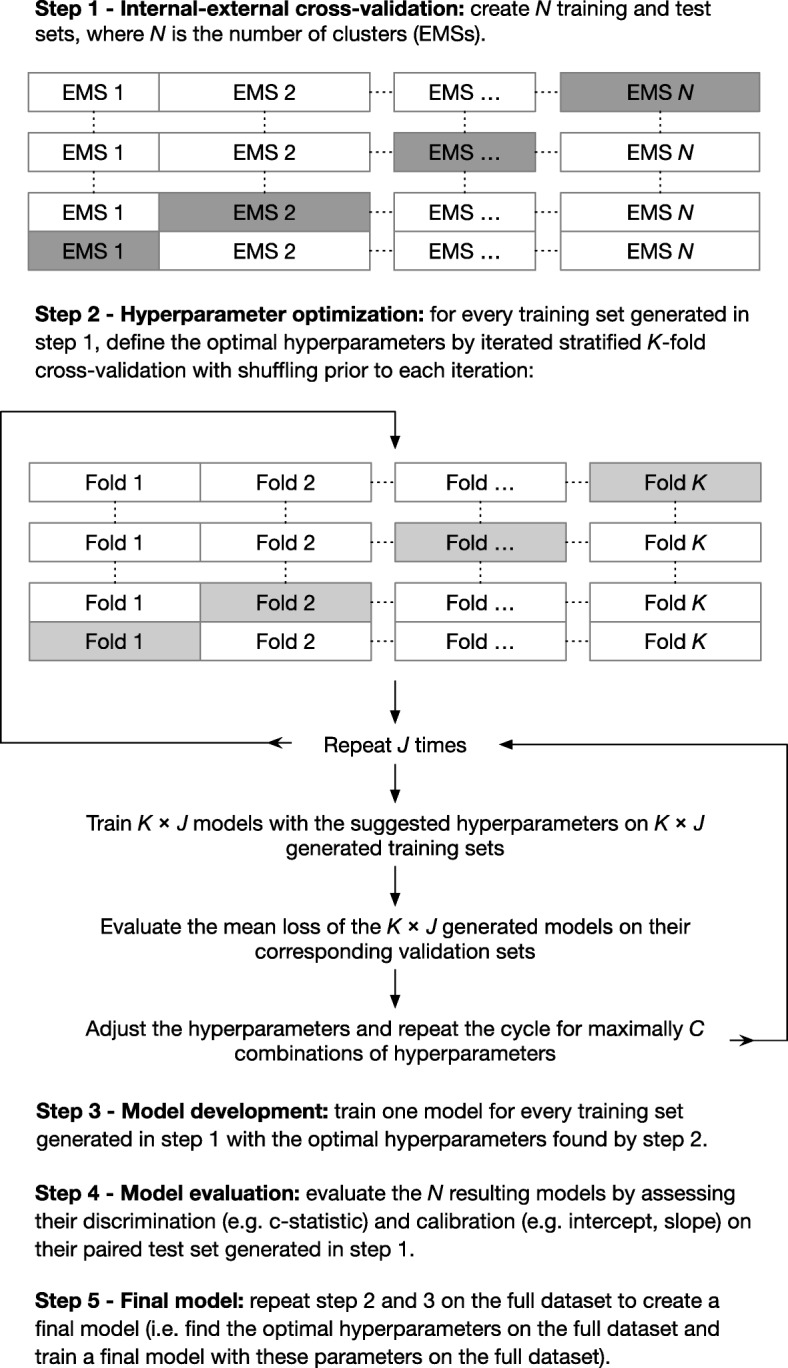
Model development methodology

The third and final step will be to construct one model based on the complete dataset. The full model development strategy is illustrated in Fig. 2.

Estimates of model discrimination (c-statistic) and calibration (intercept and slope) from all hold-out samples (i.e., the different clusters) will be pooled separately by IPD-MA for both the reference model and the newly developed model. Random effects meta-analysis models, in which the weights are based on the within- and between-cluster error variance, will be used to account for heterogeneity between the available clusters [30]. The between-study standard deviation will be reported from the IPD-MA. Restricted maximum likelihood estimation will be applied to estimate variance components, and the Hartung-Knapp-Sidik-Jonkman method will be used to derive 95% confidence intervals for the summary estimates of model performance [30].

## Discussion

Trauma systems can only reach their full potential when patients are transported to the right hospitals within the right time. Mortality rates, morbidity rates, and costs can be potentially reduced by mimizing undertriage, overtriage, and interhospital transfer rates. A prehospital triage tool is crucial to aid EMS professionals in order to achieve this goal.

The TTApp provides a digital platform that is easy to use, fast, and capable of incorporating complex prediction models, and provides the possibility for iterative improvements. The new prediction model proposed in this study protocol aims to improve predictive accuracy and generalizability through a robust model development strategy.

### Limitations

One key element of trauma systems is centralization, which should enable the most efficient use of finite resources. Centralization and its positive consequences (i.e., high-volume trauma centers) are known to lower mortality rates. One limitation of the primary outcome is the use of an ISS ≥ 16 as the reference standard for the need of specialized trauma care, since the ISS is a scale that does not perfectly correlate with resource use [17, 18]. The secondary outcome eliminates this limitation, but is not officially used to evaluate triage accuracy [1].

A second limitation is that we focus on gradient boosting decision tree and do not evaluate other prediction modeling strategies. However, we do not aim to develop a perfect prediction model (which is impossible anyhow) and believe that the size of our dataset, the restriction of unknown hyperparameters, and the implementation of regularization will prevent overfitting. Furthermore, by avoiding additional comparisons with other modeling strategies, we effectively minimize the danger of chance findings and overoptimism. Finally, it is important to realize that we chose to avoid regression analysis as the current prediction model for trauma triage (which is based on logistic regression) suffers from missing values in clinical practice, a problem that is remedied by adopting gradient boosting models.

A third limitation of this study is the use of frequentist meta-analysis methods to evaluate model performance in new settings and populations. In this regard, the estimation of between-cluster heterogeneity and prediction intervals may benefit from adopting a Bayesian approach. [31]

### Implications

The TTApp is currently implemented at multiple EMSs in the Netherlands. This existing infrastructure allows us to replace the reference model with the newly developed model if it proves to be better. A software update will then implement the new prediction model on the currently used devices, so that the new model can be used almost instantly. Higher predictive accuracy and better generalizability of the TTApp will likely lead to reduced mistriage rates and, as a consequence, lower mortality rates and less life-long disabilities. The final model will be made available as a Python object through the supplementary content.

## Conclusions

The TTApp is currently used by multiple EMSs in the Netherlands to provide EMS professionals with decision support during field triage. This study protocol outlines the methodology that will be used to construct an improved prediction model, with emphasis on high predictive accuracy and broad generalizability.

## Abbreviations

BSA: Basic Set of Ambulance Care (in Dutch, Basisset Ambulancezorg)
EMS: Emergency Medical Service
IECV: Internal-external cross-validation
IPD: Individual participant data
IPD-MA: Individual participant data meta-analysis
ISS: Injury Severity Score
LTR: Dutch National Trauma Registry (in Dutch, Landelijke Traumaregistratie)
TRIPOD: Transparent Reporting of a multivariable model for Individual Prognosis or Diagnosis
TTApp: Trauma Triage App
